# Supplementary material for: Kikwit Ebola Virus Disease Progression in the Rhesus Monkey Animal Model
Source: Viruses. 2020 Jul 14;12(7):753. doi: 10.3390/v12070753 (PMC7411891; doi:10.3390/v12070753)
Supplement: Supplementary file 1 [file viruses-12-00753-s001.pdf]

Supplementary tables (S)

Kikwit Ebola Virus Disease Progression in the Rhesus Monkey Animal Model

Richard S. Bennett [1,*,†], James Logue [1,†], David X. Liu [1], Rebecca J. Reeder [1], Krisztina B. Janosko [1], Donna L. Perry [1], Timothy K. Cooper [1], Russell Byrum [1], Danny Ragland [1], Marisa St. Claire [1], Ricky Adams [1], Tracey L. Burdette [1], Tyler M. Brady [1], Kyra Hadley [1], M. Colin Waters [1], Rebecca Shim [1], William Dowling [2], Jing Qin [3], Ian Crozier [4], Peter B. Jahrling [1,5] and Lisa E. Hensley [1]

[1] Integrated Research Facility, Division of Clinical Research, National Institute of Allergy and Infectious Diseases, National Institutes of Health, 8200 Research Plaza, Frederick, MD 27102, USA; James.Logue@som.umaryland.edu (J.L.); xianhong.liu@nih.gov (D.X.L.); rebecca.reeder@nih.gov (R.J.R.); krisztina.janosko@nih.gov (K.B.J.); donna.perry@nih.gov (D.L.P.); timothy.cooper@nih.gov (T.K.C.); byrumr@niaid.nih.gov (R.B.); raglandd@niaid.nih.gov (D.R.); stclairem@niaid.nih.gov (M.S.C.); ricky.adams@nih.gov (R.A.); tracey.burdette@nih.gov (T.L.B.); tyler.brady@nih.gov (T.M.B.); kyra.hadley@nih.gov (K.H.); mason.waters@nih.gov (M.C.W.); rebecca.shim@nih.gov (R.S.); jahrlingp@niaid.nih.gov (P.B.J.); lisa.hensley@nih.gov (L.E.H.)

[2] Research Resources Section, Office of Biodefense, Research Resources, and Translational Research/Division of Microbiology and Infectious Diseases, National Institute of Allergy and Infectious Diseases, National Institutes of Health, 5601 Fishers Lane, Rockville, MD 20892-9825, USA; william.dowling@cepi.net

[3] Biostatistics Research Branch, National Institute of Allergy and Infectious Diseases, National Institutes of Health, 5601 Fishers Lane, Rockville, MD 20892, USA; jingqin@niaid.nih.gov

[4] Clinical Monitoring Research Program Directorate, Frederick National Laboratory for Cancer Research, Frederick, MD 21702 USA; ian.crozier@nih.gov

[5] Emerging Viral Pathogens Section, National Institute of Allergy and Infectious Diseases, National Institutes of Health, 8200 Research Plaza, Frederick, MD 21702, USA

* Correspondence: Richard.Bennett@nih.gov; Tel.: +1-301-631-7257

† These authors contributed equally to this work.

Received: 24 April 2020; Accepted: 6 July 2020; Published: 14 July 2020

Supplementary tables (S)

Table S1: Animal characteristics and study group assignments.

Challenge group	Group	Animal	Age[a] (year months)	Weight (kg)	Sex
1	Uninfected Controls	NHP C1	7y 2m	7.32	Female
		NHP C2	6y 0m	4.72	Female
		NHP C3	6y 0m	8.08	Male
	Terminal Necropsy (No Manipulations)	NHP1	5y 7m	13.02	Male
		NHP2	6y 7m	6.58	Female
		NHP3	6y 10m	6.60	Female
		NHP4	5y 7m	10.02	Male
		NHP5	6y 5m	8.08	Male
		NHP6	6y 6m	5.42	Female
2	Terminal Necropsy (Routine Manipulations)	NHP7	6y 10m	5.48	Female
		NHP8	6y 0m	6.30	Female
		NHP9	6y 6m	10.98	Male
		NHP10	6y 10m	5.82	Female
		NHP11	6y 11m	9.32	Male
		NHP12	5y 4m	8.26	Male
3	Scheduled Necropsy (3 Days Post-infection)	NHP13	6y 0m	10.14	Male
		NHP14	7y 1m	6.64	Female
		NHP15	5y 11m	5.96	Female
	Scheduled Necropsy (5 Days Post-infection)	NHP16	7y 1m	5.10	Female
		NHP17	7y 0m	7.34	Female
		NHP18	6y 0m	8.04	Female
4	Scheduled Necropsy (4 Days Post-infection)	NHP19	6y 6m	6.10	Female
		NHP20	6y 5m	9.46	Male
		NHP21	6y 3m	8.10	Male
	Scheduled Necropsy (6 Days Post-infection)	NHP22	7y 0m	7.42	Female
		NHP23	6y 11m	10.86	Male
		NHP24	6y 2m	5.32	Female

[a]Age at study day 0.

Abbreviations: kg; kilogram

Table S2 Clinical Scoring System. Clinical scores were assigned to each animal at least once daily. Scores from each section were added together and animals were considered to reach clinical endpoint once that clinical score sum reached at least a 10.

Parameter	Degree of Parameter	Score
**Overall clinical appearance; signs of hemorrhage**	Normal appearance, no petechial or ecchymotic hemorrhages	0
	Facial edema, photophobia, cyanosis, prolonged coagulation profile	2
	Severe diarrhea, vomiting, dehydration, petechiae	5
	Persistent epistaxis, melena, retrobulbar hemorrhage	10
**Respiratory rate, mucous membranes (MM) color, and dyspnea (difficulty breathing/ labored respirations)**	Normal signs (Respiratory rate = 32 to 50 BPM; MM pink)	0
	Mild (Respiratory rate = 51 to 65 BPM, with slightly increased effort; MM pale pink)	2
	Moderate (Respiratory rate = 66 to 80 BPM, with obvious difficulty breathing; MM muddy pink)	7
	Severe (Respiratory rate = > 80 BPM; respirations labored; MM blue)	10
**Recumbency**	Normal – Not lying down when enter room	0
	Lying down when entering room but gets up readily when approached	5
	Lying down when entering room but gets up with stimulation at cage front	8
	Lies down; will not get up even with excessive stimulation at cage front	10
**Non-Responsiveness**	Normal – bright, alert, responsive	0
	Mild, slightly depressed; slightly decreased appetite; puts head down when personnel in room; acts disinterested when personnel in room	3
	Moderately non-responsive; very disinterested in personnel; hunched or lying down; will get up when approached or prodded	5
	Severe; completely non-responsive to noxious stimuli such as toe-pinch; will not get up despite significant prodding	10
**Core temperature of anesthetized animal (Normal in non-anesthetized macaque = 37 – 39°C = 98.6 – 102.2°F)**	>36.7°C (>98°F)	0
	36.7>X>33.8°C (98-93.3°F)	3
	<33.8°C (<93.2°F)	10
**Total clinical score**		*

Table S3 Clinical scores

Subject Group	Subject ID	Study day with clinical score collection time indicated																				
		BL-1	BL-2	0	1	2	3	4			5			6			7			8		
		E	E	E	E	M	L	E	M	L	E	M	L	E	M	L	E	M	L	E	M	L
Non-infected Control	NHP C1	0	0	0																		
	NHP C2	0	0	0																		
	NHP C3	0	0	0																		
Terminal: No Manipulations	NHP1	0	0	0	0	0	0	0			3	8	8	11								
	NHP2	0	0	0	0	0	0	0			5	3	3	3	3	8	15					
	NHP3	0	0	0	0	0	0	0			3	3	3	3	3	3	3	3	3	3	18	
	NHP4	0	0	0	0	0	0	0			3	3	3	3	3	3	3	10				
	NHP5	0	0	0	0	0	0	0			13											
	NHP6	0	0	0	0	0	0	0			3	3	3	3	3	3	3	3	3	15		
Terminal: Routine Manipulations	NHP7	0	0	0	0	0	0	3	3		3	3		3	7	7	15					
	NHP8	0	0	0	0	0	0	3	3		3	3		5	9							
	NHP9	0	0	0	0	0	0	0	3		3	3		3	5	5	5	7	7	7	12	
	NHP10	0	0	0	0	0	0	0	0		3	3		3	5	5	5	5	15			
	NHP11	0	0	0	0	0	0	3	3		3	3		3	7	14						
	NHP12	0	0	0	0	0	0	0	0		3	3		3	3	3	3	5	7	SD		
Necropsy Day 3	NHP13	0	0	0	0	0	0															
	NHP14	0	0	0	0	0	0															
	NHP15	0	0	0	0	0	0															
Necropsy Day 5	NHP16	0	0	0	0	0	0	3	3		3											
	NHP17	0	0	0	0	0	0	0	0		0											
	NHP18	0	0	0	0	0	0	0	0		3											
Necropsy Day 4	NHP19	0	0	0	0	0	0	3														
	NHP20	0	0	0	0	0	0	0														
	NHP21	0	0	0	0	0	0	0														
Necropsy Day 6	NHP22	0	0	0	0	0	0	0	3		3	3	5	7								
	NHP23	0	0	0	0	0	0	0	0		3	3	3	3								
	NHP24	0	0	0	0	0	0	0	3		3	5	5	5								

Abbreviations: E, early time check; M, midday animal check; L, late afternoon animal check; BL, Baseline. White spaces indicate times when scores were not collected.

Table S4 Rash

Subject Group	Subject ID	BL-1	BL-2	0	1	2	3	4	5	6	7	8
Non-infected Control	NHP C1											
	NHP C2											
	NHP C3											
Terminal: No Manipulations	NHP1								Face	Face		
	NHP2								Face	Face	Face	
	NHP3								Face		Face	
	NHP4									Face	Face/arms	
	NHP5											
	NHP6											Face
Terminal: Routine Manipulations	NHP7								Face	Face	Face	
	NHP8								Face	Face		
	NHP9									Face/Arms	Face	Face
	NHP10									Face/Arms	Face	
	NHP11								Arms	Face/Arms		
	NHP12										Face/Arms	SD
Necropsy Day 3	NHP13											
	NHP14											
	NHP15											
Necropsy Day 5	NHP16											
	NHP17											
	NHP18								Face			
Necropsy Day 4	NHP19											
	NHP20											
	NHP21											
Necropsy Day 6	NHP22									Face		
	NHP23											
	NHP24											

Abbreviations: BL, baseline.

Table S5 Biscuit consumption (percent)

Subject Group	Subject ID	BL-1	BL-2	0	1	2	3	4	5	6	7	8
Non-infected Control	NHP C1	56	59	42								
	NHP C2	72	67	86								
	NHP C3	72	67	56								
Terminal: No Manipulations	NHP1	100	100	100	89	86	100	0	8	0		
	NHP2	50	78	81	47	44	44	0	0	0	0	
	NHP3	50	75	11	5.6	11	39	0	0	0	0	0
	NHP4	83	89	100	67	58	31	11	0	0	3	
	NHP5	100	86	56	17	36	39	8	0			
	NHP6	50	53	50	31	28	44.	6	6	0	3	3
Terminal: Routine Manipulations	NHP7	100	100	71	83	92	21	4	4	0	0	
	NHP8	81	61	64	72	39	50	25	0	0		
	NHP9	100	100	100	83	89	86	0	8	3	0	0
	NHP10	86	50	64	56	53	75	25	0	0	0	
	NHP11	100	94	69	88	63	47	34	0	0		
	NHP12	64	75	83	89	81	81	36	0	6	0	0
Necropsy Day 3	NHP13	100	100	100	97	63	100					
	NHP14	94	100	94	47	34	66					
	NHP15	50	66	66	56	50	63					
Necropsy Day 5	NHP16	44	44	31	13	31	53	0	0			
	NHP17	56	44	63	53	41	59	28	25			
	NHP18	100	75	79	0	38	75	0	0			
Necropsy Day 4	NHP19	56	59	66	34	63	38	31				
	NHP20	100	100	97	100	100	100	89				
	NHP21	100	100	100	86	78	89	56				
Necropsy Day 6	NHP22	92	75	88	25	58	33	0	0	0		
	NHP23	100	100	97	72	92	81	72	0	0		
	NHP24	44	38	47	19	44	34	16	0	0		

Abbreviations: BL, baseline.

Table S6 Temperature data (Celsius)

Subject Group	Subject ID	BL-1	BL-2	0	1	2	3	4	5	6	7	8
Non-infected Control	NHP C1	39.8	38.8	38.3								
	NHP C2	39.4	38.3	38.7								
	NHP C3	40.1	39.8	39.6								
Terminal: No Manipulations	NHP1	38.1		39.2						39.4		
	NHP2	39.8		39.8							35.6	
	NHP3	38.1		38.6								39.4
	NHP4	38.3		39.8							39.6	
	NHP5	38.7		39.6					39.0			
	NHP6	38.7		39.1								38.7
Terminal: Routine Manipulations	NHP7	38.8	38.9	38.6			39.8		39.0		35.3	
	NHP8	38.8	38.6	38.7			39.0		39.4	38.8		
	NHP9	39.0	39.3	39.8			39.7		40.5			39.2
	NHP10	39.7	38.7	39.7			39.8		40.1		39.3	
	NHP11	39.0	38.3	39.5			40.8		40.3	39.7		
	NHP12	39.1	39.4	39.7			40.0		40.5			35.2
Necropsy Day 3	NHP13	39.5	38.1	39.7	40.0		38.6					
	NHP14	38.9	38.9	39.2	39.4		39.9					
	NHP15	39.3	39.0	39.1	39.1		40.2					
Necropsy Day 5	NHP16	39.3	38.7	39.6	39.6		40.1		39.4			
	NHP17	39.6	38.9	39.1	39.8		39.4		40.1			
	NHP18	40.0	39.4	39.4	39.7		40.1		39.8			
Necropsy Day 4	NHP19	38.6	38.5	38.8		39.1		41.0				
	NHP20	39.1	39.0	38.8		38.2		40.8				
	NHP21	39.1	39.0	39.3		38.9		39.9				
Necropsy Day 6	NHP22	38.9	39.2	40.0		39.1		40.8		38.7		
	NHP23	39.7	38.7	39.8		39.1		41.2		40.2		
	NHP24	39.5	38.7	39.3		38.8		39.7		38.7		

Abbreviations: BL, baseline.

Table S7 Viremia (RT-qPCR)

Subject Group	Subject ID	BL-1	0	1	2	3	4	5	6	7	8
Non-infected Control	NHP C1	UND	UND								
	NHP C2	UND	UND								
	NHP C3	UND	UND								
Terminal: No Manipulations	NHP1	UND							3.61E7		
	NHP2	UND								4.85E5	
	NHP3	UND									1.00E7
	NHP4	UND								1.82E6	
	NHP5	UND						1.4E5			
	NHP6	UND									1.01E6
Terminal: Routine Manipulations	NHP7	UND				2.97E3		1.02E7		2.09E7	
	NHP8	UND				628		3.84E6	1.93E7		
	NHP9	UND				105E3		5.24E5			1.52E6
	NHP10	UND				895		3.18E5		2.59E6	
	NHP11	UND				743		3.53E5	5.73E6		
	NHP12	UND				647		3.50E6			1.16E7
Necropsy Day 3	NHP13	UND		UND		77					
	NHP14	UND		UND		819					
	NHP15	UND		UND		183					
Necropsy Day 5	NHP16	UND		UND		435		1.66E5			
	NHP17	UND		UND		2.47		949			
	NHP18	UND		UND		314		1.07E6			
Necropsy Day 4	NHP19	UND			UND		4.62E5				
	NHP20	UND			UND		2.51E3				
	NHP21	UND			UND		3.23E3				
Necropsy Day 6	NHP22	UND			UND		1.36E5		4.41E7		
	NHP23	UND			UND		1.14E5		4.52E5		
	NHP24	UND			UND		3.63E4		7.65E6		

Abbreviations: BL, baseline; UND, undetected.

Table S8 Virus in oral swabs (RT-qPCR)

Subject Group	Subject ID	BL-1	BL-2	0	1	2	3	4	5	6	7	8
Non-infected Control	NHP C1	UND		UND								
	NHP C2	UND		UND								
	NHP C3	UND		UND								
Terminal: No Manipulations	NHP1	UND								4.92E5		
	NHP2	UND									8.75E6	
	NHP3	UND										6.75E6
	NHP4	UND										4.66E6
	NHP5	UND								2.0E6		
	NHP6	UND									2.44E7	
Terminal: Routine Manipulations	NHP7		UND				UND		UND		5.25E6	
	NHP8		UND				UND		2.14E5	5.51E5		
	NHP9		UND				UND		1.19E5			2.73E7
	NHP10		UND				UND		2.68E4		7.13E6	
	NHP11		UND				UND		UND	4.34E5		
	NHP12		UND				UND		UND			3,10E7
Necropsy Day 3	NHP13		UND		UND		UND					
	NHP14		UND		UND		UND					
	NHP15		UND		UND		UND					
Necropsy Day 5	NHP16	UND			UND		UND		UND			
	NHP17	UND			UND		UND		UND			
	NHP18	UND			UND		UND		UND			
Necropsy Day 4	NHP19	UND				UND		UND				
	NHP20	UND				UND		UND				
	NHP21	UND				UND		UND				
Necropsy Day 6	NHP22	UND				UND		UND				
	NHP23	UND				UND		UND				
	NHP24	UND				UND		UND				

Abbreviations: BL, baseline; UND, undetected.

Table S9 Alanine Aminotransferase (units)

Subject Group	Subject ID	BL-1	BL-2	1	2	3	4	5	6	7	8
Non-infected Control	NHP C1	31	30								
	NHP C2	36	28								
	NHP C3	46	52								
Terminal: No Manipulations	NHP1	33	42						207		
	NHP2	33	34							188	
	NHP3	26	23								177
	NHP4	54	31							330	
	NHP5	29	29					342			
	NHP6	32	31								379
Terminal: Routine Manipulations	NHP7	41	32			34		665		1261	
	NHP8	41	51			60		124	340		
	NHP9	24	34			26		68			185
	NHP10	39	49			63		102		253	
	NHP11	45	54			31		60	240		
	NHP12	44	47			47		121			740
Necropsy Day 3	NHP13	47	53	63		55					
	NHP14	40	37	47		42					
	NHP15	33	30	31		31					
Necropsy Day 5	NHP16	39	41	58		52		90			
	NHP17	29	29	39		34		42			
	NHP18	20	18	45				277			
Necropsy Day 4	NHP19	51	39		62		52				
	NHP20	30	29		52		51				
	NHP21	43	46		53		42				
Necropsy Day 6	NHP22	13	10		22		21		126		
	NHP23	30	32		41		41		143		
	NHP24	48	28		38		45		715		

Abbreviations: BL, baseline.

Table S10 Albumin

Subject Group	Subject ID	BL-1	BL-2	1	2	3	4	5	6	7	8
Non-infected Control	NHP C1	3.3	3.6								
	NHP C2	3.6	3.2								
	NHP C3	3.7	3.4								
Terminal: No Manipulations	NHP1	3.2	3.4						2		
	NHP2	4	3.9							1.9	
	NHP3	3.4	3.2								2.1
	NHP4	3.9	3.6							2.1	
	NHP5	4.1	3.9					2.4			
	NHP6	3.4	3.3								2.2
Terminal: Routine Manipulations	NHP7	2.7	2.7			2.6		2		1.6	
	NHP8	3.2	3.4			3.5		3	2.8		
	NHP9	3.5	3.5			3.4		2.7			2.2
	NHP10	3.5	3.4			3.3		2.7		2	
	NHP11	2.7	2.7			2.4		1.9	1.9		
	NHP12	3.9	3.7			3.3		3			2.3
Necropsy Day 3	NHP13	3.5	3.5	3.3		3.1					
	NHP14	3.4	3.2	3.2		3.5					
	NHP15	3.5	3.2	3.4		3.8					
Necropsy Day 5	NHP16	3.4	3.2	3.5		3.6		2.8			
	NHP17	3.7	3.5	3.5		3.7		2.9			
	NHP18	3.6	3.6	3.7				2.9			
Necropsy Day 4	NHP19	3.6	3.4		3.3		3.2				
	NHP20	3.6	3.3		3.1		3.3				
	NHP21	4.1	3.9		3.8		3.2				
Necropsy Day 6	NHP22	3.4	3.3		3.2		3.3		2.3		
	NHP23	3.7	3.8		3.6		3.5		2.9		
	NHP24	3.7	3.5		3.5		3.9		2.7		

Abbreviations: BL, baseline.

Table S11 Albumin:Globulin (Ratio)

Subject Group	Subject ID	BL-1	BL-2	1	2	3	4	5	6	7	8
Non-infected Control	NHP C1	0.94	1.16								
	NHP C2	0.92	0.89								
	NHP C3	1.16	1.10								
Terminal: No Manipulations	NHP1	0.89	1.03						0.50		
	NHP2	1.08	1.15							0.54	
	NHP3	0.79	0.89								0.54
	NHP4	0.91	1.00							0.51	
	NHP5	1.11	1.30					0.42			
	NHP6	0.92	1.00								0.59
Terminal: Routine Manipulations	NHP7	0.93	1.00			0.74		0.54		0.47	
	NHP8	0.91	1.03			1.00		0.81	0.74		
	NHP9	1.00	1.17			1.03		0.82			0.61
	NHP10	1.03	1.03			0.92		0.79		0.63	
	NHP11	0.73	0.77			0.65		0.53	0.49		
	NHP12	1.05	1.16			0.87		0.75			0.53
Necropsy Day 3	NHP13	0.97	0.95	0.92		0.97					
	NHP14	1.03	0.91	0.94		1.06					
	NHP15	1.13	0.97	0.94		1.36					
Necropsy Day 5	NHP16	0.92	0.97	0.92		1.06		0.76			
	NHP17	1.19	1.13	1.13		1.42		0.97			
	NHP18	0.9	0.88	0.88				0.78			
Necropsy Day 4	NHP19	1.03	1.03		0.97		0.89				
	NHP20	1.00	1.03		0.89		0.94				
	NHP21	1.24	1.18		1.09		0.97				
Necropsy Day 6	NHP22	0.77	0.80		0.71		0.70		0.46		
	NHP23	0.97	1.03		0.90		0.78		0.71		
	NHP24	1.12	1.13		0.97		1.03		0.82		

Abbreviations: BL, baseline.

Table S12 Alkaline Phosphatase

Subject Group	Subject ID	BL-1	BL-2	1	2	3	4	5	6	7	8
Non-infected Control	NHP C1	65	69								
	NHP C2	154	135								
	NHP C3	355	326								
Terminal: No Manipulations	NHP1	166	164						415		
	NHP2	139	130							1249	
	NHP3	127	85								522
	NHP4	256	168							1789	
	NHP5	199	183					458			
	NHP6	93	84								462
Terminal: Routine Manipulations	NHP7	63	83			136		560		659	
	NHP8	127	137			154		266	637		
	NHP9	181	152			158		261			765
	NHP10	82	81			100		155		600	
	NHP11	164	157			187		305	890		
	NHP12	395	354			333		353			1666
Necropsy Day 3	NHP13	250	206	197		183					
	NHP14	117	102	123		121					
	NHP15	190	178	189		191					
Necropsy Day 5	NHP16	106	106	115		116		143			
	NHP17	128	130	141		142		142			
	NHP18	108	94	88				348			
Necropsy Day 4	NHP19	114	124		103		144				
	NHP20	117	115		97		116				
	NHP21	322	280		271		245				
Necropsy Day 6	NHP22	153	152		152		229		510		
	NHP23	150	167		148		141		197		
	NHP24	109	126		111		135		952		

Abbreviations: BL, baseline.

Table S13 Amylase

Subject Group	Subject ID	BL-1	BL-2	1	2	3	4	5	6	7	8
Non-infected Control	NHP C1	208	191								
	NHP C2	372	353								
	NHP C3	628	657								
Terminal: No Manipulations	NHP1	351	372						732		
	NHP2	354	354							690	
	NHP3	868	904								350
	NHP4	483	437							291	
	NHP5	386	422					364			
	NHP6	238	263								210
Terminal: Routine Manipulations	NHP7	416	413			341		209		409	
	NHP8	252	225			248		167	158		
	NHP9	479	460			470		329			232
	NHP10	386	492			389		187		192	
	NHP11	351	279			299		203	418		
	NHP12	458	358			445		256			702
Necropsy Day 3	NHP13	433	406	436		371					
	NHP14	327	329	655		337					
	NHP15	291	340	274		250					
Necropsy Day 5	NHP16	229	235	369		208		142			
	NHP17	411	407	371		348		255			
	NHP18	355	307	329				139			
Necropsy Day 4	NHP19	298	253		306		244				
	NHP20	464	408		385		383				
	NHP21	349	331		347		275				
Necropsy Day 6	NHP22	456	378		431		286		370		
	NHP23	458	459		474		437		456		
	NHP24	418	384		422		315		163		

Abbreviations: BL, baseline.

Table S14 Aspartate Aminotransferase

Subject Group	Subject ID	BL-1	BL-2	1	2	3	4	5	6	7	8
Non-infected Control	NHP C1	32	27								
	NHP C2	29	31								
	NHP C3	42	41								
Terminal: No Manipulations	NHP1	30	45						502		
	NHP2	42	35							791	
	NHP3	23	22								631
	NHP4	50	33							656	
	NHP5	42	31					1396			
	NHP6	40	35								1386
Terminal: Routine Manipulations	NHP7	32	32			34		1325		O	
	NHP8	38	36			35		334	1154		
	NHP9	37	41			36		215			906
	NHP10	35	38			97		218		795	
	NHP11	36	33			30		202	641		
	NHP12	30	41			28		254			1963
Necropsy Day 3	NHP13	36	36	52		45					
	NHP14	41	38	95		45					
	NHP15	28	33	36		30					
Necropsy Day 5	NHP16	39	33	75		48		176			
	NHP17	36	38	64		49		45			
	NHP18	28	29	70				615			
Necropsy Day 4	NHP19	37	31		41		56				
	NHP20	35	32		38		40				
	NHP21	39	39		43		57				
Necropsy Day 6	NHP22	30	25		36		24		868		
	NHP23	46	45		36		62		311		
	NHP24	34	24		25		55		1590		

Abbreviations: BL, baseline; O, Outside the reference range.

Table S15 Blood Urea Nitrogen

Subject Group	Subject ID	BL-1	BL-2	1	2	3	4	5	6	7	8
Non-infected Control	NHP C1	16	19								
	NHP C2	18	19								
	NHP C3	23	25								
Terminal: No Manipulations	NHP1	20	19						31		
	NHP2	19	25							>180	
	NHP3	16	11								28
	NHP4	22	20							89	
	NHP5	25	19					95			
	NHP6	14	11								37
Terminal: Routine Manipulations	NHP7	20	22			11		37		>180	
	NHP8	12	13			10		9	27		
	NHP9	19	22			18		8			
	NHP10	20	20			17		13		22	
	NHP11	13	15			10		9	21		
	NHP12	19	27			20		15			159
Necropsy Day 3	NHP13	19	26	23		21					
	NHP14	23	22	18		19					
	NHP15	17	17	17		18					
Necropsy Day 5	NHP16	18	20	18		19		17			
	NHP17	12	17	12		18		13			
	NHP18	15	20	13				11			
Necropsy Day 4	NHP19	15	16		12		14				
	NHP20	18	19		19		18				
	NHP21	17	16		17		17				
Necropsy Day 6	NHP22	23	23		18		16		45		
	NHP23	19	17		17		14		17		
	NHP24	25	19		19		16		37		

Abbreviations: BL, baseline.

Table S16 BUN:Creatinine

Subject Group	Subject ID	BL-1	BL-2	1	2	3	4	5	6	7	8
Non-infected Control	NHP C1	26.67	23.75								
	NHP C2	18	27.14								
	NHP C3	23	31.25								
Terminal: No Manipulations	NHP1	28.57	23.75						14.09		
	NHP2	19	27.78							0	
	NHP3	22.86	15.71								21.54
	NHP4	24.44	20							19.35	
	NHP5	25	21.11					17.92			
	NHP6	15.56	13.75								16.82
Terminal: Routine Manipulations	NHP7	20	24.44			13.75		16.82		0	
	NHP8	13.33	18.57			16.67		9	19.29		
	NHP9	17.27	24.44			16.36		6.15			15.83
	NHP10	22.22	25			21.25		11.82		16.92	
	NHP11	14.44	18.75			11.11		8.18	13.13		
	NHP12	21.11	33.75			28.57		13.64			18.71
Necropsy Day 3	NHP13	23.75	23.64	38.33		26.25					
	NHP14	28.75	27.5	30		21.11					
	NHP15	18.89	24.29	34		22.5					
Necropsy Day 5	NHP16	20	20	25.71		19		14.17			
	NHP17	13.33	18.89	20		25.71		16.25			
	NHP18	25	28.57	32.5				9.17			
Necropsy Day 4	NHP19	21.43	20		17.14		12.73				
	NHP20	22.5	23.75		31.67		15				
	NHP21	24.29	26.67		28.33		15.45				
Necropsy Day 6	NHP22	28.75	28.75		25.71		16		8.33		
	NHP23	21.11	18.89		24.29		15.56		10.63		
	NHP24	41.67	31.67		31.67		20		26.43		

Abbreviations: BL, baseline.

Table S17 Calcium (mg/dL)

Subject Group	Subject ID	BL-1	BL-2	1	2	3	4	5	6	7	8
Non-infected Control	NHP C1	9.5	9.5								
	NHP C2	9.2	9.3								
	NHP C3	9.4	9.6								
Terminal: No Manipulations	NHP1	9.1	9						7.1		
	NHP2	9.8	9.9							4.9	
	NHP3	9.2	9.2								7.7
	NHP4	10	9.8							5.8	
	NHP5	9.9	9.5					6.8			
	NHP6	9.1	8.6								7.8
Terminal: Routine Manipulations	NHP7	8.8	9			9.1		7.9		5.1	
	NHP8	9.6	9.6			10		8.3	7.6		
	NHP9	9.7	9.5			9.4		7.9			7.9
	NHP10	9.2	9.1			9.2		8.3		8.1	
	NHP11	8.8	9.2			8.4		7.8	8		
	NHP12	9.6	9.5			9.6		8.4			8.8
Necropsy Day 3	NHP13	9.7	9.7	9.9		9.1					
	NHP14	9.1	9.6	9.6		9.1					
	NHP15	9.2	9.1	9.5		9.1					
Necropsy Day 5	NHP16	9.4	9.4	9.9		9.2		8.9			
	NHP17	9.5	9.6	10.1		9		9.1			
	NHP18	9.6	9.9	9.7				8.4			
Necropsy Day 4	NHP19	9.6	9.3		9.7		9.1				
	NHP20	9.6	9.5		9.8		9				
	NHP21	10.4	9.9		10		9.3				
Necropsy Day 6	NHP22	9.4	9.2		9.2		8.7		7		
	NHP23	9.8	9.6		10.2		9.6		8.6		
	NHP24	9.4	9.2		9.7		9.2		7.8		

Abbreviations: BL, baseline.

Table S18 Creatinine (mg/dL)

Subject Group	Subject ID	BL-1	BL-2	1	2	3	4	5	6	7	8
Non-infected Control	NHP C1	0.6	0.8								
	NHP C2	1	0.7								
	NHP C3	1	0.8								
Terminal: No Manipulations	NHP1	0.7	0.8						2.2		
	NHP2	1	0.9							13.5	
	NHP3	0.7	0.7								1.3
	NHP4	0.9	1							4.6	
	NHP5	1	0.9					5.3			
	NHP6	0.9	0.8								2.2
Terminal: Routine Manipulations	NHP7	1	0.9			0.8		2.2		9.7	
	NHP8	0.9	0.7			0.6		1	1.4		
	NHP9	1.1	0.9			1.1		1.3			2.4
	NHP10	0.9	0.8			0.8		1.1		1.3	
	NHP11	0.9	0.8			0.9		1.1	1.6		
	NHP12	0.9	0.8			0.7		1.1			8.5
Necropsy Day 3	NHP13	0.8	1.1	0.6		0.8					
	NHP14	0.8	0.8	0.6		0.9					
	NHP15	0.9	0.7	0.5		0.8					
Necropsy Day 5	NHP16	0.9	1	0.7		1		1.2			
	NHP17	0.9	0.9	0.6		0.7		0.8			
	NHP18	0.6	0.7	0.4				1.2			
Necropsy Day 4	NHP19	0.7	0.8		0.7		1.1				
	NHP20	0.8	0.8		0.6		1.2				
	NHP21	0.7	0.6		0.6		1.1				
Necropsy Day 6	NHP22	0.8	0.8		0.7		1		5.4		
	NHP23	0.9	0.9		0.7		0.9		1.6		
	NHP24	0.6	0.6		0.6		0.8		1.4		

Abbreviations: BL, baseline.

Table S19 Gamma-glutamyl Transpeptidase (μL)

Subject Group	Subject ID	BL-1	BL-2	1	2	3	4	5	6	7	8
Non-infected Control	NHP C1	71	65								
	NHP C2	74	63								
	NHP C3	122	133								
Terminal: No Manipulations	NHP1	46	45						78		
	NHP2	38	40							379	
	NHP3	49	52								140
	NHP4	93	76							309	
	NHP5	73	71					270			
	NHP6	46	44								195
Terminal: Routine Manipulations	NHP7	37	36			46		207		266	
	NHP8	65	59			71		89	259		
	NHP9	78	65			61		57			237
	NHP10	48	46			52		49		155	
	NHP11	95	90			95		83	194		
	NHP12	68	59			69		76			315
Necropsy Day 3	NHP13	100	90	89		77					
	NHP14	61	63	65		61					
	NHP15	56	56	60		55					
Necropsy Day 5	NHP16	59	52	64		60		54			
	NHP17	56	57	66		61		52			
	NHP18	32	29	31				42			
Necropsy Day 4	NHP19	46	43		43		43				
	NHP20	69	61		66		61				
	NHP21	96	91		105		78				
Necropsy Day 6	NHP22	70	63		71		68		151		
	NHP23	63	65		65		75		119		
	NHP24	67	58		64		63		157		

Abbreviations: BL, baseline.

Table S20 Globulin (g/dL)

Subject Group	Subject ID	BL-1	BL-2	1	2	3	4	5	6	7	8
Non-infected Control	NHP C1	3.5	3.1								
	NHP C2	3.9	3.6								
	NHP C3	3.2	3.1								
Terminal: No Manipulations	NHP1	3.6	3.3						4		
	NHP2	3.7	3.4							3.5	
	NHP3	4.3	3.6								3.9
	NHP4	4.3	3.6							4.1	
	NHP5	3.7	3					5.7			
	NHP6	3.7	3.3								3.7
Terminal: Routine Manipulations	NHP7	2.9	2.7			3.5		3.7		3.4	
	NHP8	3.5	3.3			3.5		3.7	3.8		
	NHP9	3.5	3			3.3		3.3			3.6
	NHP10	3.4	3.3			3.6		3.4		3.2	
	NHP11	3.7	3.5			3.7		3.6	3.9		
	NHP12	3.7	3.2			3.8		4			4.3
Necropsy Day 3	NHP13	3.6	3.7	3.6		3.2					
	NHP14	3.3	3.5	3.4		3.3					
	NHP15	3.1	3.3	3.6		2.8					
Necropsy Day 5	NHP16	3.7	3.3	3.8		3.4		3.7			
	NHP17	3.1	3.1	3.1		2.6		3			
	NHP18	4	4.1	4.2				3.7			
Necropsy Day 4	NHP19	3.5	3.3		3.4		3.6				
	NHP20	3.6	3.2		3.5		3.5				
	NHP21	3.3	3.3		3.5		3.3				
Necropsy Day 6	NHP22	4.4	4.1		4.5		4.7		5		
	NHP23	3.8	3.7		4		4.5		4.1		
	NHP24	3.3	3.1		3.6		3.8		3.3		

Abbreviations: BL, baseline.

Table S21 Glucose (mg/dL)

Subject Group	Subject ID	BL-1	BL-2	1	2	3	4	5	6	7	8
**Non-infected Control**	**NHP C1**	63	63								
	**NHP C2**	79	63								
	**NHP C3**	70	70								
**Terminal: No Manipulations**	**NHP1**	69	73						76		
	**NHP2**	62	90							37	
	**NHP3**	60	68								72
	**NHP4**	64	62							108	
	**NHP5**	56	67					67			
	**NHP6**	57	60								50
**Terminal: Routine Manipulations**	**NHP7**	55	51			83		59		49	
	**NHP8**	64	62			69		71	74		
	**NHP9**	112	54			106		92			48
	**NHP10**	61	58			67		75		69	
	**NHP11**	64	57			66		66	81		
	**NHP12**	67	66			76		91			12
**Necropsy Day 3**	**NHP13**	76	96	70		69					
	**NHP14**	63	66	68		72					
	**NHP15**	79	70	76		54					
**Necropsy Day 5**	**NHP16**	57	58	72		83		45			
	**NHP17**	71	71	74		67		71			
	**NHP18**	79	75	100				53			
**Necropsy Day 4**	**NHP19**	69	68		78		57				
	**NHP20**	66	61		76		71				
	**NHP21**	77	69		66		67				
**Necropsy Day 6**	**NHP22**	67	61		75		86		48		
	**NHP23**	59	57		66		87		93		
	**NHP24**	64	60		73		81		46		

Abbreviations: BL, baseline.

Table S22 Total Bilirubin (mg/dL)

Subject Group	Subject ID	BL-1	BL-2	1	2	3	4	5	6	7	8
Non-infected Control	NHP C1	0.4	0.4								
	NHP C2	0.3	0.3								
	NHP C3	0.4	0.4								
Terminal: No Manipulations	NHP1	0.4	0.4						0.7		
	NHP2	0.4	0.4							4.8	
	NHP3	0.5	0.4								0.6
	NHP4	0.5	0.4							1.8	
	NHP5	0.5	0.4					0.5			
	NHP6	0.4	0.4								0.8
Terminal: Routine Manipulations	NHP7	0.4	0.4			0.4		0.6		2.8	
	NHP8	0.3	0.4			0.4		0.5	0.9		
	NHP9	0.4	0.5			0.4		0.4			1.3
	NHP10	0.4	0.5			0.4		0.6		1.1	
	NHP11	0.4	0.4			0.4		0.5	1.5		
	NHP12	0.4	0.5			0.4		0.5			2.5
Necropsy Day 3	NHP13	0.4	0.4	0.5		0.4					
	NHP14	0.4	0.4	0.4		0.4					
	NHP15	0.5	0.4	0.4		0.4					
Necropsy Day 5	NHP16	0.5	0.4	0.5		0.5		0.5			
	NHP17	0.5	0.4	0.4		0.5		0.5			
	NHP18	0.3	0.3	0.4				0.5			
Necropsy Day 4	NHP19	0.4	0.6		0.5		0.5				
	NHP20	0.4	0.4		0.4		0.4				
	NHP21	0.5	0.4		0.4		0.5				
Necropsy Day 6	NHP22	0.4	0.5		0.5		0.8		0.7		
	NHP23	0.4	0.5		0.4		0.5		0.4		
	NHP24	0.6	0.5		0.5		0.7		0.9		

Abbreviations: BL, baseline.

Table S23 Total Protein (g/dL)

Subject Group	Subject ID	BL-1	BL-2	1	2	3	4	5	6	7	8
Non-infected Control	NHP C1	6.8	6.7								
	NHP C2	7.5	6.8								
	NHP C3	6.9	6.5								
Terminal: No Manipulations	NHP1	6.8	6.7						6		
	NHP2	7.7	7.3							5.4	
	NHP3	7.7	6.8								6
	NHP4	8.2	7.2							6.2	
	NHP5	7.8	6.9					8.1			
	NHP6	7.1	6.6								5.9
Terminal: Routine Manipulations	NHP7	5.6	5.4			6.1		5.7		5	
	NHP8	6.7	6.7			7		6.7	6.6		
	NHP9	7	6.5			6.7		6			5.8
	NHP10	6.9	6.7			6.9		6.1		5.2	
	NHP11	6.4	6.2			6.1		5.5	5.8		
	NHP12	7.6	6.9			7.1		7			6.6
Necropsy Day 3	NHP13	7.1	7.2	6.9		6.3					
	NHP14	6.7	6.7	6.6		6.8					
	NHP15	6.6	6.5	7		6.6					
Necropsy Day 5	NHP16	7.1	6.5	7.3		7		6.5			
	NHP17	6.8	6.6	6.6		6.3		5.9			
	NHP18	7.6	7.7	7.9				6.6			
Necropsy Day 4	NHP19	7.1	6.7		6.7		6.8				
	NHP20	7.2	6.5		6.6		6.8				
	NHP21	7.4	7.2		7.3		6.5				
Necropsy Day 6	NHP22	7.8	7.4		7.7		8		7.3		
	NHP23	7.5	7.5		7.6		8		7		
	NHP24	7	6.6		7.1		7.7		6		

Abbreviations: BL, baseline.

Table S24 Basophil Count (10^3 cells/μL)

Subject Group	Subject ID	BL-1	BL-2	1	2	3	4	5	6	7	8
Non-infected Control	NHP C1	0.01	0.02								
	NHP C2	0.02	0.01								
	NHP C3	0.01	0								
Terminal: No Manipulations	NHP1	0.01	0.01						0.02		
	NHP2	0.01	0.01							0.01	
	NHP3	0.02	0.01								0
	NHP4	0.01	0.01							0.09	
	NHP5	0.02	0.01					0.03			
	NHP6	0.01	0								0.04
Terminal: Routine Manipulations	NHP7	0.02	0.01			0.01		0.01		0	
	NHP8	0.01	0.02			0.02		0.01	0.01		
	NHP9	0.01	0.01			0.02		0			0.07
	NHP10	0	0			0.01		0		0.02	
	NHP11	0.01	0.01			0.01		0.01	0		
	NHP12	0.01	0.01			0.02		0.01			0.47
Necropsy Day 3	NHP13	0.01	0.01	0.03		0.01					
	NHP14	0.01	0.01	0.01		0.01					
	NHP15	0.03	0.03	0.04		0.02					
Necropsy Day 5	NHP16	0.02	0.05	0.06		0.02		0.03			
	NHP17	0	0.01	0.02		0.01		0.02			
	NHP18	0	0.01	0.02				0.02			
Necropsy Day 4	NHP19	0.03	0.01		0.05		0.04				
	NHP20	0.02	0.01		0.01		0.01				
	NHP21	0.01	0.04		0.03		0.03				
Necropsy Day 6	NHP22	0.02	0.02		0.03		0.01		0.01		
	NHP23	0.02	0.01		0		0		0		
	NHP24	0.01	0.01		0.02		0.01		0.01		

Abbreviations: BL, baseline.

Table S25 Eosinophil Count (10^3 cells/μL)

Subject Group	Subject ID	BL-1	BL-2	1	2	3	4	5	6	7	8
Non-infected Control	NHP C1	0.06	0.04								
	NHP C2	0	0								
	NHP C3	0.06	0.05								
Terminal: No Manipulations	NHP1	0	0.05						0		
	NHP2	0	0.04							0	
	NHP3	0.05	0.04								0.01
	NHP4	0.07	0.1							0.02	
	NHP5	0.04	0.02					0.05			
	NHP6	0.1	0.1								0.03
Terminal: Routine Manipulations	NHP7	0.02	0			0.01		0.01		0.02	
	NHP8	0.21	0.03			0.11		0.02	0.02		
	NHP9	0.15	0			0.02		0.05			0.01
	NHP10	0.09	0.06			0.03		0.01		0	
	NHP11	0.13	0.05			0.01		0.01	0.02		
	NHP12	0.05	0			0.01		0			0.04
Necropsy Day 3	NHP13	0.25	0	0.12		0.02					
	NHP14	0.07	0.19	0.15		0.25					
	NHP15	0.04	0.15	0.04		0					
Necropsy Day 5	NHP16	0.23	0.84	0.17		0.02		0.01			
	NHP17	0.02	0	0		0		0			
	NHP18	0.02	0.01	0.02				0.02			
Necropsy Day 4	NHP19	0.07	0.02		0.01		0				
	NHP20	0.19	0.06		0.03		0				
	NHP21	0.07	0.03		0.02		0				
Necropsy Day 6	NHP22	0.03	0.01		0.01		0		0.01		
	NHP23	0.03	0.03		0		0		0.04		
	NHP24	0.02	0.01		0		0		0		

Abbreviations: BL, baseline.

Table S26 Hemoglobin (g/dL)

Subject Group	Subject ID	BL-1	BL-2	1	2	3	4	5	6	7	8
Non-infected Control	NHP C1	10.8	10								
	NHP C2	11.4	10.4								
	NHP C3	11.1	10.2								
Terminal: No Manipulations	NHP1	12.6	12.2						11.3		
	NHP2	12.4	12							11.6	
	NHP3	12.5	12								12
	NHP4	13.3	12.6							13.3	
	NHP5	13.4	13.4					10.8			
	NHP6	13.2	12.1								11
Terminal: Routine Manipulations	NHP7	10.9	8.9			11.7		9.8		8.7	
	NHP8	10.6	10.4			11.2		10.1	10.6		
	NHP9	12.6	11.1			12		10.6			12.1
	NHP10	9.9	9.3			9.7		9		9.7	
	NHP11	12.6	12.1			12.2		10.3	12.9		
	NHP12	12.8	11.4			12.2		10.6			11.9
Necropsy Day 3	NHP13	11.6	11.6	11.3		10.8					
	NHP14	11.3	11.3	11.8		12.2					
	NHP15	11.3	10.5	11.5		10.6					
Necropsy Day 5	NHP16	11.5	11.1	12.2		11.1		10.3			
	NHP17	11.5	11.2	11.7		11.1		10.8			
	NHP18	12.2	12.2	12				10.5			
Necropsy Day 4	NHP19	12.2	10.7		11		10.9				
	NHP20	12.9	12		12.3		12.4				
	NHP21	12.5	11.8		12.4		12.6				
Necropsy Day 6	NHP22	10.8	9.7		11.7		9.7		9.9		
	NHP23	13	11.8		12.5		12.3		11.7		
	NHP24	11	9.9		10.7		11.1		10.1		

Abbreviations: BL, baseline.

Table S27 Hematocrit (%)

Subject Group	Subject ID	BL-1	BL-2	1	2	3	4	5	6	7	8
Non-infected Control	NHP C1	33.6	31.3								
	NHP C2	36.8	33.9								
	NHP C3	34.1	31.8								
Terminal: No Manipulations	NHP1	40.4	39.3						35.5		
	NHP2	38.7	37.6							35.1	
	NHP3	39.8	37.4								35.6
	NHP4	41.1	39							40.5	
	NHP5	42.5	42					31.6			
	NHP6	41.7	37.4								34.5
Terminal: Routine Manipulations	NHP7	32.2	27.5			35.8		29.9		26.1	
	NHP8	32.9	32.6			35		30.5	31.3		
	NHP9	37.3	34.1			36.8		32			35.4
	NHP10	32.2	29.4			31.6		28.8		29.7	
	NHP11	38.5	37.2			37.4		31	39.6		
	NHP12	38.6	34.3			37.2		32.2			37.6
Necropsy Day 3	NHP13	36.9	36.3	35.1		33.6					
	NHP14	34.3	34.5	35.2		36.6					
	NHP15	35.6	33	36.8		32.7					
Necropsy Day 5	NHP16	36.8	35.7	38.4		34.9		31.9			
	NHP17	36.4	35.5	36.5		34.5		33.5			
	NHP18	38.6	38.3	37.5				31.8			
Necropsy Day 4	NHP19	37.2	32.9		33.8		33.6				
	NHP20	39.2	36.6		37.6		37.2				
	NHP21	38.6	37.2		38.7		38.1				
Necropsy Day 6	NHP22	33.5	30.5		35.3		30.3		29.9		
	NHP23	40.4	37.1		39.0		38		35.6		
	NHP24	33.5	30.5		32.4		33.7		29.6		

Abbreviations: BL, baseline.

Table S28 Lymphocyte Counts (10^3 cells/μL)

Subject Group	Subject ID	BL-1	BL-2	1	2	3	4	5	6	7	8
Non-infected Control	**NHP C1**	2.87	2.94								
	**NHP C2**	1.93	2.51								
	**NHP C3**	2.82	3.17								
Terminal: No Manipulations	**NHP1**	1.93	2.35						1.96		
	**NHP2**	2.81	2.98							2.41	
	**NHP3**	4.27	3.51								1.46
	**NHP4**	2.3	2.24							1.65	
	**NHP5**	1.98	1.99					5.49			
	**NHP6**	2.25	2.99								1.6
Terminal: Routine Manipulations	**NHP7**	1.17	2.02			1.4		1.97		2.8	
	**NHP8**	3.55	3.75			3.15		1.62	2.18		
	**NHP9**	2.23	2.77			2.15		1.49			1.43
	**NHP10**	2.05	2.27			1.83		1.13		1.4	
	**NHP11**	3.63	3.41			2.26		1.51	1.08		
	**NHP12**	1.72	1.32			1.42		0.73			3.93
Necropsy Day 3	**NHP13**	2.52	2.98	2.04		1.2					
	**NHP14**	1.82	2.4	2.14		1.3					
	**NHP15**	2.03	2.21	2.38		1.35					
Necropsy Day 5	**NHP16**	2.07	2.39	2.77		1.62		0.96			
	**NHP17**	0.76	1.1	1.28		1.02		1.76			
	**NHP18**	1.72	2.17	1.71				1.04			
Necropsy Day 4	**NHP19**	6.05	4.67		5.27		1.4				
	**NHP20**	3.11	1.99		2.08		1.46				
	**NHP21**	2.5	1.82		2.41		1.23				
Necropsy Day 6	**NHP22**	4.91	3.17		2.69		1.6		1.23		
	**NHP23**	2.4	2.12		2.2		1.17		1.15		
	**NHP24**	2.66	1.93		2.69		1.07		1.81		

Abbreviations: BL, baseline.

Table S29 Mean Corpuscular Hemoglobin (pico)

Subject Group	Subject ID	BL-1	BL-2	1	2	3	4	5	6	7	8
Non-infected Control	NHP C1	21.2	21.6								
	NHP C2	24.2	24.4								
	NHP C3	22.3	22.1								
Terminal: No Manipulations	NHP1	22	21.9						22.5		
	NHP2	21.6	21.7							21.9	
	NHP3	24.9	24.8								25
	NHP4	26	26.1							26	
	NHP5	23.9	24.3					24.6			
	NHP6	23.8	23.8								23.5
Terminal: Routine Manipulations	NHP7	26.8	26.3			26.1		26.2		27.1	
	NHP8	22.7	22.5			22.4		22.5	22.4		
	NHP9	26	25.3			24.9		25			24.7
	NHP10	23.9	24.3			24.2		23.9		23.9	
	NHP11	23.2	23.2			23.1		23.6	23		
	NHP12	24.9	25.2			24.8		25			24.6
Necropsy Day 3	NHP13	24.4	24.5	25		24.6					
	NHP14	26.2	26.5	27.1		26.6					
	NHP15	25.4	25.9	25.5		26.2					
Necropsy Day 5	NHP16	24.1	24.1	24.4		24.3		24.4			
	NHP17	25.1	25.1	25.3		25.3		25.3			
	NHP18	23.6	23.6	24				24.3			
Necropsy Day 4	NHP19	25.5	26		25.9		25.9				
	NHP20	23.5	23.7		23.6		23.8				
	NHP21	24.9	24.9		25		25.5				
Necropsy Day 6	NHP22	25.1	25.3		25.9		25.2		24.9		
	NHP23	22.4	22.4		22.3		22.5		22.4		
	NHP24	25.2	25.3		25.5		25.3		25.7		

Abbreviations: BL, baseline.

Table S30 Mean Corpuscular Hemoglobin Concentration (g/dL)

Subject Group	Subject ID	BL-1	BL-2	1	2	3	4	5	6	7	8
Non-infected Control	NHP C1	32.1	31.9								
	NHP C2	31	30.7								
	NHP C3	32.6	32.1								
Terminal: No Manipulations	NHP1	31.2	31						31.8		
	NHP2	32	31.9							33	
	NHP3	31.4	32.1								33.7
	NHP4	32.4	32.3							32.8	
	NHP5	31.5	31.9					34.2			
	NHP6	31.7	32.4								31.9
Terminal: Routine Manipulations	NHP7	33.9	32.4			32.7		32.8		33.3	
	NHP8	32.2	31.9			32		33.1	33.9		
	NHP9	33.8	32.6			32.6		33.1			34.2
	NHP10	30.7	31.6			30.7		31.3		32.7	
	NHP11	32.7	32.5			32.6		33.2	32.6		
	NHP12	33.2	33.2			32.8		32.9			31.6
Necropsy Day 3	NHP13	31.4	32	32.2		32.1					
	NHP14	32.9	32.8	33.5		33.3					
	NHP15	31.7	31.8	31.3		32.4					
Necropsy Day 5	NHP16	31.3	31.1	31.8		31.8		32.3			
	NHP17	31.6	31.5	32.1		32.2		32.2			
	NHP18	31.6	31.9	32				33			
Necropsy Day 4	NHP19	32.8	32.5		32.5		32.4				
	NHP20	32.9	32.8		32.7		33.3				
	NHP21	32.4	31.7		32		33.1				
Necropsy Day 6	NHP22	32.2	31.8		33.1		32		33.1		
	NHP23	32.2	31.8		32.1		32.4		32.9		
	NHP24	32.8	32.5		33		32.9		34.1		

Abbreviations: BL, baseline.

Table S31 Mean Corpuscular Volume (femto)

Subject Group	Subject ID	BL-1	BL-2	1	2	3	4	5	6	7	8
Non-infected Control	NHP C1	66	67.5								
	NHP C2	78.1	79.6								
	NHP C3	68.6	69								
Terminal: No Manipulations	NHP1	70.4	70.6						70.7		
	NHP2	67.3	67.9							66.2	
	NHP3	79.3	77.3								74.2
	NHP4	80.4	80.7							79.3	
	NHP5	75.9	76.2					72			
	NHP6	75.3	73.6								73.6
Terminal: Routine Manipulations	NHP7	79.3	81.4			79.9		79.9		81.3	
	NHP8	70.4	70.6			70.1		67.9	66		
	NHP9	76.9	77.9			76.3		75.5			72.4
	NHP10	77.8	77			78.8		76.4		73.2	
	NHP11	71	71.3			71		71.1	70.7		
	NHP12	75.1	75.9			75.8		75.9			77.7
Necropsy Day 3	NHP13	77.7	76.7	77.7		76.5					
	NHP14	79.6	80.8	80.9		79.9					
	NHP15	80	81.3	81.6		80.9					
Necropsy Day 5	NHP16	77.1	77.6	76.6		76.4		75.6			
	NHP17	79.5	79.6	78.8		78.6		78.5			
	NHP18	74.7	74.1	75				73.6			
Necropsy Day 4	NHP19	77.7	79.9		79.5		79.8				
	NHP20	71.4	72.3		72		71.4				
	NHP21	76.7	78.5		78		77				
Necropsy Day 6	NHP22	77.9	79.6		78.3		78.7		75.3		
	NHP23	69.5	70.4		69.6		69.5		68.1		
	NHP24	76.7	78		77.1		76.9		75.3		

Abbreviations: BL, baseline.

Table S32 Mean Platelet Volume (femto)

Subject Group	Subject ID	BL-1	BL-2	1	2	3	4	5	6	7	8
Non-infected Control	NHP C1	11.1	10.3								
	NHP C2	11.6	11.2								
	NHP C3	9.5	9.3								
Terminal: No Manipulations	NHP1	11.7	11.9						10.4		
	NHP2	11.3	11.1							0	
	NHP3	11.5	11.2								10.6
	NHP4	12.2	11.5							0	
	NHP5	11.1	10.7					0			
	NHP6	10.8	8.5								9.7
Terminal: Routine Manipulations	NHP7	10.2	10.8			10.9		11		9.4	
	NHP8	10.5	9.8			10.2		10.4	10.2		
	NHP9	11.5	10.9			11.2		11.5			10.1
	NHP10	10.8	10.3			10.9		10.9		10.2	
	NHP11	11.1	10.3			10.5		11.2	10		
	NHP12	10.8	10.1			10.6		10.8			0
Necropsy Day 3	NHP13	10.7	10.4	10.2		10.2					
	NHP14	11.5	11	11.2		12.4					
	NHP15	12.2	11.3	11.7		11.9					
Necropsy Day 5	NHP16	9.9	9.2	9.7		9.8		10.1			
	NHP17	10.2	9.7	9.9		9.9		10			
	NHP18	11.4	11.1	11.2				10.7			
Necropsy Day 4	NHP19	11.4	10.8		11		11.7				
	NHP20	9.6	8.6		9.1		9.8				
	NHP21	10	9.5		9.6		10.4				
Necropsy Day 6	NHP22	11	10.5		11.3		11.7		10.3		
	NHP23	9.3	9.3		8.9		8.9		9.4		
	NHP24	11.1	10.6		10.8		10.8		10.6		

Abbreviations: BL, baseline.

Table S33 Monocyte Counts (10^3/μL)

Subject Group	Subject ID	BL-1	BL-2	1	2	3	4	5	6	7	8
Non-infected Control	NHP C1	0.4	0.37								
	NHP C2	0.23	0.24								
	NHP C3	0.49	0.41								
Terminal: No Manipulations	NHP1	0.24	0.34						0.02		
	NHP2	0.33	0.37							0.01	
	NHP3	0.4	0.2								0.05
	NHP4	0.21	0.25							0.19	
	NHP5	0.33	0.24					0.11			
	NHP6	0.51	0.34								0.11
Terminal: Routine Manipulations	NHP7	0.44	0.69			1.36		0.15		0.04	
	NHP8	0.19	0.45			0.78		0.04	0.11		
	NHP9	0.24	0.67			0.73		0.03			0.22
	NHP10	0.18	0.26			0.35		0.08		0.03	
	NHP11	0.34	0.43			0.97		0.06	0		
	NHP12	0.24	0.33			0.6		0.13			1.33
Necropsy Day 3	NHP13	0.23	0.3	0.41		1.02					
	NHP14	0.19	0.15	0.2		0.63					
	NHP15	0.25	0.29	0.45		0.77					
Necropsy Day 5	NHP16	0.3	0.5	0.32		0.76		0.02			
	NHP17	0.19	0.42	0.45		0.59		2.08			
	NHP18	0.34	0.45	0.4				0.01			
Necropsy Day 4	NHP19	0.82	0.59		0.63		0.64				
	NHP20	0.63	0.52		0.44		1.13				
	NHP21	0.27	0.34		0.47		1.13				
Necropsy Day 6	NHP22	0.31	0.52		0.28		0.53		0.01		
	NHP23	0.35	0.5		0		0.32		0.72		
	NHP24	0.19	0.27		0.14		0.18		0.05		

Abbreviations: BL, baseline.

Table S34 Neutrophil Count (10^3 cells/μL)

Subject Group	Subject ID	BL-1	BL-2	1	2	3	4	5	6	7	8
Non-infected Control	NHP C1	3.12	2.95								
	NHP C2	4.23	4.09								
	NHP C3	3.64	3.29								
Terminal: No Manipulations	NHP1	10.5	4.59						15.29		
	NHP2	1.78	2.44							9.65	
	NHP3	8.08	3.97								6.43
	NHP4	2.86	2.83							0.68	
	NHP5	3.51	2.42					12.08			
	NHP6	4.71	3.36								4.47
Terminal: Routine Manipulations	NHP7	4.52	7.66			7.66		4.92		2.96	
	NHP8	1.86	5.3			7.93		7.78	2.48		
	NHP9	3.27	14.92			10.2		7.46			7.02
	NHP10	1.83	5.68			6.4		4.12		2.89	
	NHP11	4.16	10.2			14.15		16.65	6.48		
	NHP12	1.94	11.46			6.69		6.04			5.04
Necropsy Day 3	NHP13	2.65	0	3.95		7.63					
	NHP14	1.59	1.73	0.81		3.37					
	NHP15	2.95	2.69	3.08		8.62					
Necropsy Day 5	NHP16	2.04	3.84	1.43		10.7		6.26			
	NHP17	3.44	6.54	3.85		10.81		12.72			
	NHP18	2.36	5.17	2.94				8.24			
Necropsy Day 4	NHP19	3.96	3.57		3.28		22.01				
	NHP20	2.68	3.11		1.75		13.28				
	NHP21	1.42	2		3.51		17.62				
Necropsy Day 6	NHP22	2.15	7.21		2.43		19.6		5.07		
	NHP23	3.96	8.98		5.2		13.39		6.42		
	NHP24	2.23	3.38		2.1		13.02		4.1		

Table S35 Percent Basophil (%)

Subject Group	Subject ID	BL-1	BL-2	1	2	3	4	5	6	7	8
Non-infected Control	NHP C1	0.2	0.3								
	NHP C2	0.3	0.1								
	NHP C3	0.1	0								
Terminal: No Manipulations	NHP1	0.1	0.1						0.1		
	NHP2	0.2	0.2							0.1	
	NHP3	0.2	0.1								0
	NHP4	0.2	0.2							3.4	
	NHP5	0.3	0.2					0.2			
	NHP6	0.1	0								0.6
Terminal: Routine Manipulations	NHP7	0.3	0.1			0.1		0.1		0	
	NHP8	0.2	0.2			0.2		0.1	0.2		
	NHP9	0.2	0.1			0.2		0			0.8
	NHP10	0	0			0.1		0		0.5	
	NHP11	0.1	0.1			0.1		0.1	0		
	NHP12	0.3	0.1			0.2		0.1			4.3
Necropsy Day 3	NHP13	0.2	0.2	0.5		0.1					
	NHP14	0.3	0.2	0.3		0.2					
	NHP15	0.6	0.6	0.7		0.2					
Necropsy Day 5	NHP16	0.4	0.7	1.3		0.2		0.4			
	NHP17	0	0.1	0.4		0.1		0.1			
	NHP18	0	0.1	0.4				0.2			
Necropsy Day 4	NHP19	0.3	0.1		0.5		0.2				
	NHP20	0.3	0.2		0.2		0.1				
	NHP21	0.2	0.9		0.5		0.1				
Necropsy Day 6	NHP22	0.3	0.2		0.6		0		0.2		
	NHP23	0.3	0.1		0.1		0		0		
	NHP24	0.2	0.2		0.4		0.1		0.2		

Abbreviations: BL, baseline.

Table S36 Percent Eosinophil (%)

Subject Group	Subject ID	BL-1	BL-2	1	2	3	4	5	6	7	8
Non-infected Control	NHP C1	0.9	0.6								
	NHP C2	0	0								
	NHP C3	0.9	0.7								
Terminal: No Manipulations	NHP1	0	0.7						0		
	NHP2	0	0.7							0	
	NHP3	0.4	0.5								0.1
	NHP4	1.3	1.8							0.8	
	NHP5	0.7	0.4					0.3			
	NHP6	1.3	1.5								0.5
Terminal: Routine Manipulations	NHP7	0.3	0			0.1		0.1		0.3	
	NHP8	3.6	0.3			0.9		0.2	0.4		
	NHP9	2.5	0			0.2		0.6			0.1
	NHP10	2.2	0.7			0.3		0.2		0	
	NHP11	1.6	0.4			0.1		0.1	0.3		
	NHP12	1.3	0			0.1		0			0.4
Necropsy Day 3	NHP13	4.4	0	1.8		0.2					
	NHP14	1.9	4.2	4.5		4.5					
	NHP15	0.8	2.8	0.7		0					
Necropsy Day 5	NHP16	4.9	11	3.6		0.2		0.1			
	NHP17	0.5	0	0		0		0			
	NHP18	0.5	0.1	0.4				0.2			
Necropsy Day 4	NHP19	0.6	0.2		0.1		0				
	NHP20	2.9	1.1		0.7		0				
	NHP21	1.6	0.7		0.3		0				
Necropsy Day 6	NHP22	0.4	0.1		0.2		0		0.2		
	NHP23	0.4	0.3		0.0		0		0.5		
	NHP24	0.4	0.2		0		0		0		

Abbreviations: BL, baseline.

Table S37 Percent Lymphocyte (%)

Subject Group	Subject ID	BL-1	BL-2	1	2	3	4	5	6	7	8
Non-infected Control	NHP C1	44.4	46.5								
	NHP C2	30.1	36.6								
	NHP C3	40.2	45.8								
Terminal: No Manipulations	NHP1	15.2	32						11.3		
	NHP2	57	51							20	
	NHP3	33.3	45.4								18.4
	NHP4	42.2	41.3							62.7	
	NHP5	33.7	42.5					30.9			
	NHP6	29.7	44								25.6
Terminal: Routine Manipulations	NHP7	19	19.5			13.4		27.9		48.1	
	NHP8	61	39.3			26.3		17.1	45.4		
	NHP9	37.8	15.1			16.4		16.5			16.3
	NHP10	49.4	27.4			21.2		21.2		32.3	
	NHP11	43.9	24.2			13		8.3	14.2		
	NHP12	43.4	10.1			16.2		10.6			36.4
Necropsy Day 3	NHP13	44.5	55.1	31.1		12.1					
	NHP14	49.5	53.6	64.7		23.4					
	NHP15	38.3	41.2	39.7		12.5					
Necropsy Day 5	NHP16	44.4	31.4	58.3		12.3		13.2			
	NHP17	17.2	13.6	22.9		8.2		10.6			
	NHP18	38.7	27.8	33.6				11.1			
Necropsy Day 4	NHP19	55.4	52.7		57		5.8				
	NHP20	46.9	35		48.3		9.2				
	NHP21	58.5	43		37.4		6.1				
Necropsy Day 6	NHP22	66.2	29		49.4		7.4		19.4		
	NHP23	35.5	18.2		27.4		7.9		13.8		
	NHP24	52.1	34.5		54.3		7.5		30.3		

Abbreviations: BL, baseline.

Table S38 Percent Monocyte (%)

Subject Group	Subject ID	BL-1	BL-2	1	2	3	4	5	6	7	8
Non-infected Control	NHP C1	6.2	5.9								
	NHP C2	3.6	3.5								
	NHP C3	7	5.9								
Terminal: No Manipulations	NHP1	1.9	4.6						0.1		
	NHP2	6.7	6.3							0.1	
	NHP3	3.1	2.6								0.6
	NHP4	3.9	4.6							7.2	
	NHP5	5.6	5.1					0.6			
	NHP6	6.7	5								1.8
Terminal: Routine Manipulations	NHP7	7.1	6.6			13		2.1		0.7	
	NHP8	3.3	4.7			6.5		0.4	2.3		
	NHP9	4.1	3.6			5.6		0.3			2.5
	NHP10	4.3	3.1			4.1		1.5		0.7	
	NHP11	4.1	3			5.6		0.3	0.4		
	NHP12	6.1	2.5			6.9		1.9			12.3
Necropsy Day 3	NHP13	4.1	5.5	6.3		10.3					
	NHP14	5.2	3.3	6		11.3					
	NHP15	4.7	5.4	7.5		7.2					
Necropsy Day 5	NHP16	6.4	6.6	6.7		5.8		0.3			
	NHP17	4.3	5.2	8		4.7		12.5			
	NHP18	7.7	5.8	7.9				0.1			
Necropsy Day 4	NHP19	7.5	6.7		6.8		2.7				
	NHP20	9.5	9.1		10.2		7.1				
	NHP21	6.3	8		7.3		5.6				
Necropsy Day 6	NHP22	4.2	4.8		5.1		2.4		0.2		
	NHP23	5.2	4.3		6.5		2.2		8.6		
	NHP24	3.7	4.8		2.8		1.3		0.8		

Abbreviations: BL, baseline.

Table S39 Percent Neutrophil (%)

Subject Group	Subject ID	BL-1	BL-2	1	2	3	4	5	6	7	8
Non-infected Control	NHP C1	3.12	2.95								
	NHP C2	4.23	4.09								
	NHP C3	3.64	3.29								
Terminal: No Manipulations	NHP1	10.5	4.59						15.29		
	NHP2	1.78	2.44							9.65	
	NHP3	8.08	3.97								6.43
	NHP4	2.86	2.83							0.68	
	NHP5	3.51	2.42					12.08			
	NHP6	4.71	3.36								4.47
Terminal: Routine Manipulations	NHP7	4.52	7.66			7.66		4.92		2.96	
	NHP8	1.86	5.3			7.93		7.78	2.48		
	NHP9	3.27	14.92			10.2		7.46			7.02
	NHP10	1.83	5.68			6.4		4.12		2.89	
	NHP11	4.16	10.2			14.15		16.65	6.48		
	NHP12	1.94	11.46			6.69		6.04			5.04
Necropsy Day 3	NHP13	2.65	0	3.95		7.63					
	NHP14	1.59	1.73	0.81		3.37					
	NHP15	2.95	2.69	3.08		8.62					
Necropsy Day 5	NHP16	2.04	3.84	1.43		10.7		6.26			
	NHP17	3.44	6.54	3.85		10.81		12.72			
	NHP18	2.36	5.17	2.94				8.24			
Necropsy Day 4	NHP19	3.96	3.57		3.28		22.01				
	NHP20	2.68	3.11		1.75		13.28				
	NHP21	1.42	2		3.51		17.62				
Necropsy Day 6	NHP22	2.15	7.21		2.43		19.6		5.07		
	NHP23	3.12	2.95								
	NHP24	4.23	4.09								

Abbreviations: BL, baseline.

Table S40 Percent Reticulocyte (%)

Subject Group	Subject ID	BL-1	BL-2	1	2	3	4	5	6	7	8
Non-infected Control	NHP C1	1.34	1.65								
	NHP C2	1.15	1.79								
	NHP C3	0.47	0.76								
Terminal: No Manipulations	NHP1	0.94	0.79						0.26		
	NHP2	0.81	1.09							0.1	
	NHP3	0.81	0.84								0.13
	NHP4	0.84	1.01							0.14	
	NHP5	1.33	0.88					0.15			
	NHP6	1.12	0.7								0.07
Terminal: Routine Manipulations	NHP7	0.32	1.16			0.79		0.51		0.13	
	NHP8	1.32	1.29			0.68		0.57	0.13		
	NHP9	0.9	1.17			0.62		0.25			0
	NHP10	1.04	0.36			1.23		0.64		0.1	
	NHP11	0.68	0.69			0.95		0.68	0.17		
	NHP12	1.21	0.92			0.97		0.37			0.11
Necropsy Day 3	NHP13	0.89	0.7	0.69		0.69					
	NHP14	1.18	1.3	1.64		1.16					
	NHP15	1.19	1.73	1.23		1.16					
Necropsy Day 5	NHP16	1.21	0.72	0.66		0.78		0.42			
	NHP17	0.9	0.97	1.11		0.64		0.68			
	NHP18	1.94	1.81	2.41				0.81			
Necropsy Day 4	NHP19	0.65	0.61		0.54		0.37				
	NHP20	1.45	1.38		1.41		1.02				
	NHP21	1.26	1.07		0.94		0.73				
Necropsy Day 6	NHP22	0.7	0.56		0.51		0.31		0.11		
	NHP23	0.98	1.35		1.2		0.58		0.18		
	NHP24	0.57	1.26		0.98		0.39		0.17		

Abbreviations: BL, baseline.

Table S41 Platelet Counts (10^3 cells/μL)

Subject Group	Subject ID	BL-1	BL-2	1	2	3	4	5	6	7	8
Non-infected Control	NHP C1	319	332								
	NHP C2	267	316								
	NHP C3	271	308								
Terminal: No Manipulations	NHP1	393	278						165		
	NHP2	125	159							29	
	NHP3	387	335								61
	NHP4	240	236							33	
	NHP5	337	306					100			
	NHP6	274	362								51
Terminal: Routine Manipulations	NHP7	257	352			263		141		102	
	NHP8	328	343			293		118	69		
	NHP9	265	302			240		119			49
	NHP10	216	201			195		93		29	
	NHP11	347	409			325		164	67		
	NHP12	330	307			293		149			50
Necropsy Day 3	NHP13	316	345	257		244					
	NHP14	360	413	343		233					
	NHP15	256	295	216		192					
Necropsy Day 5	NHP16	339	293	315		277		154			
	NHP17	254	258	257		232		169			
	NHP18	308	337	333				87			
Necropsy Day 4	NHP19	312	332		267		135				
	NHP20	399	442		412		329				
	NHP21	325	319		341		221				
Necropsy Day 6	NHP22	277	271		225		189		65		
	NHP23	365	356		354		253		155		
	NHP24	284	298		288		236		110		

Abbreviations: BL, baseline.

Table S42 Platelet Distribution Width (femto)

Subject Group	Subject ID	BL-1	BL-2	1	2	3	4	5	6	7	8
Non-infected Control	NHP C1	12.3	11.1								
	NHP C2	12.4	11.3								
	NHP C3	10	9.4								
Terminal: No Manipulations	NHP1	12.5	13.6						14.3		
	NHP2	12.5	13.2								
	NHP3	12.4	12.4								14.2
	NHP4	14.1	13.9								
	NHP5	12.9	12								
	NHP6	11.9	8.6								11.2
Terminal: Routine Manipulations	NHP7	10.6	11.1			11.8		12.8		10.9	
	NHP8	11.1	11			11		13.3	15.1		
	NHP9	12.4	11.8			12.4		13.3			16.6
	NHP10	11.4	11.1			12.8		12.9		14.3	
	NHP11	11.7	11.1			11.5		12.4	12.2		
	NHP12	11.6	10.9			11.4		11.6			
Necropsy Day 3	NHP13	11	10.9	10.7		10.5					
	NHP14	12.2	11.4	11.9		13.6					
	NHP15	13.9	12.1	13.4		14.2					
Necropsy Day 5	NHP16	10.3	9.1	10.2		10.4		10.7			
	NHP17	10.6	10.1	10.2		9.9		10.5			
	NHP18	12.4	11.8	11.6				12.4			
Necropsy Day 4	NHP19	12.2	11.4		11.9		13.6				
	NHP20	10.2	8.6		9.1		10.2				
	NHP21	9.8	9.6		9.8		10.6				
Necropsy Day 6	NHP22	12.4	11.7		13.2		13.5		15.1		
	NHP23	9.7	9.1		8.8		9.3		10.5		
	NHP24	11.8	11.2		11.9		11.8		12.2		

Abbreviations: BL, baseline.

Table S43 Platelet – Large Cell Ratio (%)

Subject Group	Subject ID	BL-1	BL-2	1	2	3	4	5	6	7	8
Non-infected Control	NHP C1	34.9	28.3								
	NHP C2	38.6	36.1								
	NHP C3	21.4	18.9								
Terminal: No Manipulations	NHP1	38.1	37.9						30.9		
	NHP2	35.6	33.3								
	NHP3	36.9	35.3								30
	NHP4	42.2	37.6								
	NHP5	34.9	30.6								
	NHP6	29.1	13.3								23.2
Terminal: Routine Manipulations	NHP7	27	31.4			33.3		32.5		21.3	
	NHP8	29.3	23.2			26.8		28.9	29.7		
	NHP9	37.3	33			36.2		37.8			28.3
	NHP10	33.3	28.4			34.1		31.9		30.4	
	NHP11	33.6	27.4			29.8		34.2	26.7		
	NHP12	32.9	26.3			30.2		31.5			
Necropsy Day 3	NHP13	31	28.3	27.6		27.8					
	NHP14	38.7	34.1	35.7		42.5					
	NHP15	42.9	36.2	38.2		41.3					
Necropsy Day 5	NHP16	24.7	17.8	22.8		23.5		26.2			
	NHP17	26.6	22.8	24.5		23.9		24.1			
	NHP18	36.7	34.5	35.8				32.2			
Necropsy Day 4	NHP19	36.9	32.3		34.2		38.5				
	NHP20	21.9	13.3		17.6		23				
	NHP21	24	20.3		22.6		28.2				
Necropsy Day 6	NHP22	34	30		35.4		38.8		30.9		
	NHP23	19.3	18.4		15.5		16.4		21.3		
	NHP24	35.1	30.8		32.3		32.3		29.4		

Abbreviations: BL, baseline.

Table S44 Plateletcrit (%)

Subject Group	Subject ID	BL-1	BL-2	1	2	3	4	5	6	7	8
Non-infected Control	NHP C1	0.35	0.34								
	NHP C2	0.31	0.36								
	NHP C3	0.26	0.29								
Terminal: No Manipulations	NHP1	0.46	0.33						0.17		
	NHP2	0.14	0.18								
	NHP3	0.44	0.37								0.06
	NHP4	0.29	0.27								
	NHP5	0.38	0.33								
	NHP6	0.3	0.31								0.05
Terminal: Routine Manipulations	NHP7	0.26	0.38			0.29		0.15		0.1	
	NHP8	0.34	0.33			0.3		0.12	0.07		
	NHP9	0.3	0.33			0.27		0.14			0.05
	NHP10	0.23	0.21			0.21		0.1		0.03	
	NHP11	0.38	0.42			0.34		0.18	0.07		
	NHP12	0.36	0.31			0.31		0.16			
Necropsy Day 3	NHP13	0.34	0.36	0.26		0.25					
	NHP14	0.41	0.46	0.38		0.29					
	NHP15	0.31	0.33	0.25		0.23					
Necropsy Day 5	NHP16	0.34	0.27	0.31		0.27		0.15			
	NHP17	0.26	0.25	0.25		0.23		0.17			
	NHP18	0.35	0.38	0.37				0.09			
Necropsy Day 4	NHP19	0.36	0.36		0.29		0.16				
	NHP20	0.38	0.38		0.38		0.32				
	NHP21	0.32	0.3		0.33		0.23				
Necropsy Day 6	NHP22	0.31	0.28		0.25		0.22		0.07		
	NHP23	0.34	0.33		0.3		0.23		0.15		
	NHP24	0.32	0.32		0.31		0.26		0.12		

Abbreviations: BL, baseline.

Table S45 Reticulocyte Counts (10^6 cells/μL)

Subject Group	Subject ID	BL-1	BL-2	1	2	3	4	5	6	7	8
Non-infected Control	NHP C1	0.0682	0.0766								
	NHP C2	0.0542	0.0763								
	NHP C3	0.0234	0.035								
Terminal: No Manipulations	NHP1	0.054	0.044						0.0131		
	NHP2	0.0466	0.0604							0.0053	
	NHP3	0.0407	0.0407								0.0062
	NHP4	0.0429	0.0488							0.0072	
	NHP5	0.0745	0.0485					0.0066			
	NHP6	0.062	0.0356								0.0033
Terminal: Routine Manipulations	NHP7	0.013	0.0392			0.0354		0.0191		0.0042	
	NHP8	0.0616	0.0596			0.0339		0.0256	0.0062		
	NHP9	0.0436	0.0512			0.0299		0.0106			
	NHP10	0.0431	0.0138			0.0493		0.0241		0.0041	
	NHP11	0.0369	0.036			0.0501		0.0296	0.0095		
	NHP12	0.0622	0.0416			0.0476		0.0157			0.0053
Necropsy Day 3	NHP13	0.0423	0.0331	0.0312		0.0303					
	NHP14	0.0509	0.0555	0.0713		0.0531					
	NHP15	0.053	0.0702	0.0555		0.0469					
Necropsy Day 5	NHP16	0.0577	0.0331	0.0331		0.0356		0.0177			
	NHP17	0.0412	0.0433	0.0514		0.0281		0.029			
	NHP18	0.1003	0.0936	0.1205				0.035			
Necropsy Day 4	NHP19	0.0311	0.0251		0.023		0.0156				
	NHP20	0.0796	0.0698		0.0736		0.0531				
	NHP21	0.0634	0.0507		0.0466		0.0361				
Necropsy Day 6	NHP22	0.0301	0.0214		0.023		0.0119		0.0044		
	NHP23	0.0569	0.0711		0.1		0.0317		0.0094		
	NHP24	0.0249	0.0493		0.0412		0.0171		0.0067		

Abbreviations: BL, baseline.

Table S46 White Blood Cell Counts (10^3 cells/μL)

Subject Group	Subject ID	BL-1	BL-2	1	2	3	4	5	6	7	8
Non-infected Control	NHP C1	6.46	6.32								
	NHP C2	6.41	6.85								
	NHP C3	7.02	6.92								
Terminal: No Manipulations	NHP1	12.68	7.34						17.29		
	NHP2	4.93	5.84							12.08	
	NHP3	12.82	7.73								7.95
	NHP4	5.45	5.43							2.63	
	NHP5	5.88	4.68					17.76			
	NHP6	7.58	6.79								6.25
Terminal: Routine Manipulations	NHP7	6.17	10.38			10.44		7.06		5.82	
	NHP8	5.82	9.55			11.99		9.47	4.8		
	NHP9	5.9	18.37			13.12		9.03			
	NHP10	4.15	8.27			8.62		5.34		4.34	
	NHP11	8.27	14.1			17.4		18.24	7.61		
	NHP12	3.96	13.12			8.74		6.91			10.81
Necropsy Day 3	NHP13	5.66	5.41	6.55		9.88					
	NHP14	3.68	4.48	3.31		5.56					
	NHP15	5.3	5.37	5.99		10.76					
Necropsy Day 5	NHP16	4.66	7.62	4.75		13.12		7.28			
	NHP17	4.41	8.07	5.6		12.43		16.58			
	NHP18	4.44	7.81	5.09				9.33			
Necropsy Day 4	NHP19	10.93	8.86		9.24		24.09				
	NHP20	6.63	5.69		4.31		15.88				
	NHP21	4.27	4.23		6.44		20.01				
Necropsy Day 6	NHP22	7.42	10.93		5.44		21.74		6.33		
	NHP23	6.76	11.64		8		14.88		8.33		
	NHP24	5.11	5.6		4.95		14.28		5.97		

Abbreviations: BL, baseline.

© 2020 by the authors. Submitted for possible open access publication under the terms and conditions of the Creative Commons Attribution (CC BY) license (http://creativecommons.org/licenses/by/4.0/).
